# Dietary Fat Intake and Risk of Gastric Cancer: A Meta-Analysis of Observational Studies

**DOI:** 10.1371/journal.pone.0138580

**Published:** 2015-09-24

**Authors:** Jun Han, Yi Jiang, Xiao Liu, Qingyang Meng, Qiulei Xi, Qiulin Zhuang, Yusong Han, Ying Gao, Qiurong Ding, Guohao Wu

**Affiliations:** 1 The Clinical Nutrition Center of Shanghai, Department of General Surgery, Zhongshan Hospital of Fudan University, Shanghai, China; 2 Nursing Department, Nanjing Maternity and Child Health Care Hospital Affiliated to Nanjing Medical University, Nanjing, Jiangsu Province, China; 3 Institute for Nutritional Sciences, Shanghai Institutes for Biological Sciences, Chinese Academy of Sciences, Shanghai, China; Tufts University, UNITED STATES

## Abstract

**Background and Objectives:**

Consumption of dietary fat has been reported to be associated with gastric cancer risk, but the results of epidemiologic studies remain inconsistent. We conducted a meta-analysis to summarize the evidence regarding the association between dietary fat intake and gastric cancer risk.

**Methods:**

A comprehensive search of PubMed and EMBASE was performed to identify observational studies providing quantitative estimates between dietary fat and gastric cancer risk. Random effects model was used to calculate the summary relative risk(SRR) in the highest versus lowest analysis. Categorical dose-response analysis was conducted to quantify the association between dietary fat intake and gastric cancer risk. Heterogeneity among studies was evaluated using I^2^ and tau2(between study variance)statistics. Subgroup analysis and publication bias analysis were also performed.

**Results:**

Twenty-two articles were included in the meta-analysis. The SRR for gastric cancer was 1.18 for individuals with highest intake versus lowest intake of total fat (95% confidence interval [CI]: 0.999–1.39; n = 28; *P*< 0.001; tau^2^ = 0.12; I^2^ = 69.5%, 95% CI: 55%-79%) and 1.08 with a daily increase in total fat intake (20 g/d) (95%CI: 1.02–1.14; n = 6; *P* = 0.09; tau^2^ = 0.002; I^2^ = 46.8%, 95% CI: 0%-79%). Positive association between saturated fat intake (SRR = 1.31; 95%CI: 1.09–1.58;n = 18;*P*<0.001; tau^2^ = 0.08; I^2^ = 60.6%, 95% CI: 34%-76%), inverse association between polyunsaturated fat intake (SRR = 0.77; 95%CI: 0.65–0.92; n = 16; *P* = 0.003; tau^2^ = 0.06; I^2^ = 56.2%, 95% CI: 23%-75%) and vegetable fat intake (SRR = 0.55; 95%CI: 0.41–0.74; n = 4;*P* = 0.12; tau^2^ = 0.04; I^2^ = 48.6%, 95% CI: 0%-83%), and no association between monounsaturated fat intake (SRR = 1.00; 95%CI: 0.79–1.25; n = 14; *P*< 0.001; tau^2^ = 0.10; I^2^ = 63.0%, 95% CI: 34%-79%) and animal fat intake (SRR = 1.10; 95%CI: 0.90–1.33; n = 6; *P* = 0.13;tau^2^ = 0.02; I^2^ = 42.0%, 95% CI: 0%-70%) and gastric cancer risk were observed.

**Conclusions:**

Our results suggest that intake of total fat is potentially positively associated with gastric cancer risk, and specific subtypes of fats account for different effects. However, these findings should be confirmed by further well-designed cohort studieswith detailed dietary assessments and strict control of confounders.

## Introduction

Gastric cancer, which account for about 10% of annual cancer-related deaths, remains one of the most common malignant tumors worldwide, especially in developing countries, such as EasternAsia, Eastern Europe, and South America[1]. Development of gastric cancer is a complex and multifactorial process. Epidemiological studies have suggested that *Helicobacter pylori* infection and dietary factors play important roles in the etiology of gastric cancer[1, 2].A distinction has been further observed between tumors arising in the proximal cardiac region(gastriccardia adenocarcinoma,GCA) and those arising in distal region (gastric non-cardia adenocarcinoma, GNCA)[3].For example, *Helicobacter pylori* infection was reported to be positively associated with the riskof GNCAbut not ofGCA[4], while overweight and obesity seem to be associated with increased risk of GCA but not ofGNCA[5].However, an expert panel,convened by the World Cancer Research Fund andthe American Institute for Cancer Research, pointed out that no dietary factorcan be convincingly proved to be risk factors for gastric cancer[6]. In spite of that, consumption of salty food, salted preserved food, red meat, and processed meat is generally thought to increase the risk of gastric cancer[7–9], while consumption of freshfruits, vegetables, and antioxidant vitamins may reduce the risk of gastric cancer[10–13]. Overall, the results of relevant studies on the effects of dietary factors on gastric cancer are inconsistent and need further investigation.

Dietary fat has been reported to be associated with various malignant tumors, such as breast cancer, colorectal cancer, pancreatic cancer, and prostate cancer [14–17].However, similar to the effect of other dietary factors, the association between dietary fat and risk of these cancers remains controversial[18, 19]. Numerous epidemiologic studies have also evaluated the contribution of dietary fatto the risk of gastric cancer. Although some case-control studies reported that high intake of dietary fat could increase the risk of gastric cancer[20, 21], some other case-control studies reported null and even inverse effect of dietary fat ongastric cancer[22, 23]. As far as we know,only one cohort study (the NIH-AARP Diet and Health study) was conducted to evaluate the association between dietary fat intake and gastric cancer risk, however, no significant association was observed [24]. Considering possible different effects may exist between specific subtypes of fat (such as saturated fat, monounsaturated fat, and polyunsaturated fat,) on gastric cancer,it is thusmore reasonable to separately analyze the effect of specific subtypes of fat.So far,very few epidemiologic studies have reported the association between intake of specific subtypes of fat and gastric cancer risk, and the existed ones gave inconsistent results.

Given the inconsistent results of previous observational studies, we performed this meta-analysis to summarize the evidence regarding the association between dietary fat intake and gastric cancer risk.

## Methods

### Literature search and study identification

A computerized literature search of PubMed and EMBASE databases was conducted to indentify relevant literatures published up to March 2015 by two independent investigators (Han and Jiang). We used mesh words and text words (“gastric neoplasm (cancer)” OR “stomach neoplasm (cancer)”) AND (“diet” OR “dietary” OR “nutrient” OR “fat” OR “saturated fat” OR “unsaturated fat” OR “polyunsaturated fat” OR “monounsaturated fat” OR “animal fat” OR “vegetable fat”) to identify relevant studies. Reference lists of review articles and retrieved articles were also reviewed for additional relevant publications.

The eligibility criteria of the studies were as follows:*1*) study designed as case-control or cohortstudy,*2*) study evaluated the association between intake of dietary fat and gastric cancer risk,*3*) study reported odds ratio (OR)or relative risk (RR) and 95% confidence intervals(CI)according to the highest versus the lowest intake of dietary fat or data sufficient to calculate them, and*4*) study was published as a full paper in English. When the studies used the same data series, only the study with most complete results or with largest population was included.

### Data extraction and quality assessment

Two authors (Han and Jiang)independently extracted data from eligible studies using standardized forms (S1 Table). Disagreements were solved by discussion with coauthors. The following items were extracted from each study: first author’s lastname, publication year, study location (country),study design type (case-control study or cohort study), number of cases, number of controls(or cohort size), dietary assessment tool, method of segregation according to dietary fat intake (tertile, quartile or quintile), fat subtypes, subgroups,RR (orOR)with their 95% CIs for the highest versus lowest category of fat intake, and adjusted factors in the analysis. If a study reported two different results adjusted for a single factor and multivariate factors, we only recorded the results adjusted for multivariate factors.

A 9-star system on the basis of the Newcastle-Ottawa Scale (NOS) was used to assess the study quality[25].A total score of 7 or greater was defined as highquality study and a total score of 6 or smaller was defined as lowquality study.

### Statistical analysis

All statistical analyses were performed with Stata version 11.0 (Stata, College Station, Texas, USA). In this analysis, we assumed OR to be comparable with the RR. For studies that only reported results for GCA and GNCA, men and women, or intestinal type and diffuse type separately, we considered them as independent data obtained from different studies.If no more than 3 studies reportedthe association between a specific subtype of fat and gastric cancer risk,we did not summary the overall effect of it.

Firstly, we conducted a meta-analysis to compare the highest category with the lowest category intake of dietary fat.DerSimonian-Laird random effects model[26], accounting for heterogeneity among studies, was used to calculate summary relative risk (SRR) and 95%CIs. The possible heterogeneity among studies was examined by using the I^2^ andtau2(between study variance)statistics[27]. Sources of heterogeneity were assessed by subgroup analyses and meta-regression analyses[28]. Subgroup analyses were performed according to gastric cancer subtype (GCA, GNCA, and mixed), geographic area (North America, Europe, and Asia and others), the number of gastric cancer cases, study design type(cohort study, population-based case-control study,and hospital-based case-control study), dietary assessment methods (health habits and history questionnaire (HHHQ), food frequency questionnaire (FFQ> 100 items AND FFQ≤ 100 items), and study quality (NOS scores). Sensitivity analyses were also conducted to estimate the influence of each study on the summary results by repeating the random effects meta-analysis after omitting one study at a time.The probability of publication bias was evaluated by visual inspection of asymmetry in funnel plots and Egger’s test and Begg’stest[29, 30]. And*P* value <0.05 was considered to be of significant publication bias.

Next, to assess the dose-response relationship between intake of dietary fat and gastric cancer risk, we conducted a meta-analysis of dose-response categorical data using the method proposed by Greenland and Orsini et al (2-stage GLST in Stata)[31, 32]. Studies were included for dose-response analysis only if they reported data for the distribution of cases and controls(orperson-time) across at least three 3 categories of exposure (dietary fat intake). Medians for each category of intake levels were required in dose-response analysis. If medians were not reported, midpoint data of each category was considered to be medians. If the highest category and the lowest category were open-ended, we assumed that it had the same amplitude as the closest category. For this analysis, the result for total fat was presented per 20 g/d increment in dose-response analysis. A restricted cubic splines model with 3 knots was used to evaluate the potential nonlinear association between dietary fat intake and gastric cancer risk.

## Results

### Literature search, study characteristics and quality assessment

**Fig 1:**
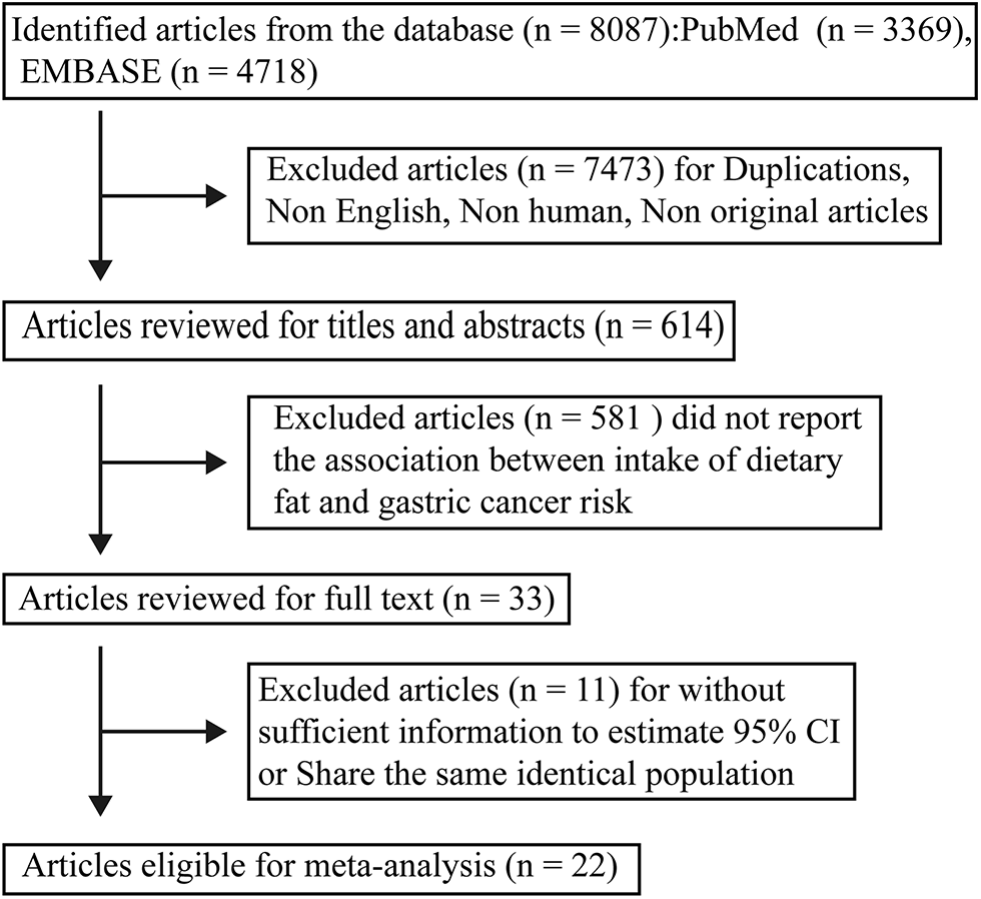
Flow chart of study selection.

We identified 8087 articles after searching PubMed and EMBASE database. Titles and abstracts of 614 articles were reviewed after removing duplicate articles, non-original articles, non-human articles, and non-English articles. Full texts of 33 articles were reviewed after excluding 581 articles with no reports of the association between dietary fat intake and gastric cancer risk. Out of these33 articles, 6 articles were excluded because they shared the same population with another 2 articles[33–38], 5 articles were excluded as they did not report 95% CI or data were not sufficient to calculate the 95% CI[39–43]. Finally, 22 articles were included in this meta-analysis(Fig 1)[20–24, 44–60].

Characteristics of the included studies are shown in S1 Table. Only one cohort study consisting of 494,978 participants and 955 gastric cancer cases was included in this meta-analysis. Of the 21 case-control studies involving 7672 cases and 20,100 controls, 6 were hospital-based and 15 were population-based. Out of 22 studies, 21, 14, 11, 12, 5, and 4 studies have investigated the associations between the gastric cancer risk and intake of total fat, saturated fat, monounsaturated fat, polyunsaturated fat, animal fat, and vegetable fat, respectively. Seven articles only separately reported results for GCA and GNCA, for men and women, or for intestinal type and diffuse type. We considered each separate report as an independent study. One study reported not only the overall result but also the separate result for GCA and GNCA[60], and we recorded all results in order to get a more accurate result in subgroup analysis. Besides, few studies have reported the results of a specific fatty acid (such astrans fat, n-3 fatty acid, oleic acid, linoleic acid, linolenic acid), we did not summarize the results of these studies. One study reported ORs according to two different time periods (during adolescence and 20 years prior to interview), and we only recorded the ORs of 20 years prior to interview[51].

The quality scores of included studies ranged from 4 to 9 based on the NOSscore system. The one cohort study and 12 case-control studies were considered to be high quality studies(NOSscore≥7), whereas 9 other case-control studies were considered to be low quality studies (NOSscore <7).

### Total fat consumption

**Fig 2:**
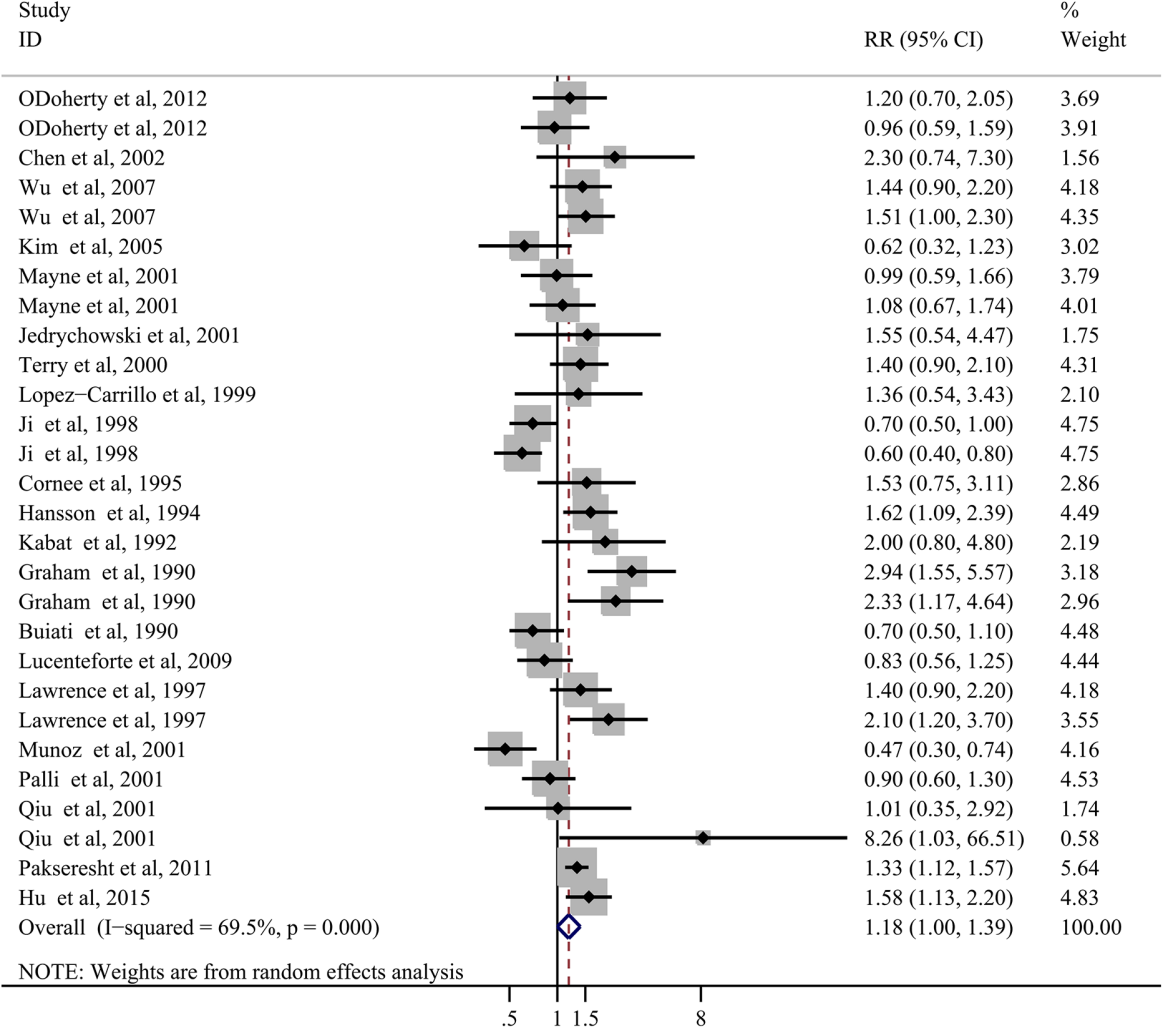
Forest plots of total fat intake and gastric cancer risk based on highest versus lowest analysis.

Twenty-one articles with 28 studies have investigated the association between total fat intake and gastric cancer risk. The SRR for the highest compared with lowest analysis was 1.18 (95% CI: 0.999–1.39; Fig 2) by the random effects model, with a high heterogeneity (*P*< 0.001; tau^2^ = 0.12; I^2^ = 69.5%, 95% CI: 55%-79%).

Subgroup analyses were conducted to examine thestability of the pooled RRs. The results indicated that a positive association between the total fat intake and gastric cancer risk was observed in studies of GNCA patients (SRR = 1.31; 95% CI: 1.14–1.51; n = 6;*P* = 0.49; I^2^ = 0.0%), studies located in North America(SRR = 1.47; 95% CI: 1.24–1.75; n = 13;*P* = 0.18; I^2^ = 26.3%), population-based case-control studies (SRR = 1.23; 95% CI: 1.01–1.50; n = 18;*P*< 0.001; I^2^ = 71.2%), studies using HHHQ (SRR = 1.69; 95% CI: 1.21–2.36; n = 3;*P* = 0.46; I^2^ = 0.00%), and studies with a small sample size(SRR = 1.53; 95% CI: 1.14–2.07; n = 13;*P* = 0.01; I^2^ = 53.8%) or low quality(SRR = 1.54; 95% CI: 1.15–1.89; n = 11;*P* = 0.01; I^2^ = 56.9%). As shown in Table 1, no evidence of a positive association between total fat intake and gastric cancer risk was observed in other subgroups.

**Table 1:** Subgroup analyses of dietary fat intake and gastric cancer risk, highest versus lowest.

		Total fat				Saturated fat				Monounsaturated fat				Polyunsaturated fat			
		N.	SRR (95% CI)	I^2^ (%)	*P*	N.	SRR (95% CI)	I^2^ (%)	*P*	N.	SRR (95% CI)	I^2^ (%)	*P*	N.	SRR (95% CI)	I^2^ (%)	*P*
Anatomic locations	GCA	5	1.23 (1.00–1.50)	0.0%	0.79	3	1.20 (0.95–1.52)	0.0%	0.40	2	1.15 (0.88–1.52)	0.0%	0.39	3	0.98 (0.79–1.20)	0.0%	0.43
	GNCA	6	1.31 (1.14–1.51)	0.0%	0.49	4	1.43 (1.16–1.76)	0.0%	0.44	2	1.15 (0.68–1.93)	71.6%	0.06	3	0.85 (0.67–1.09)	38.9%	0.19
	Mixed	18	1.14 (0.88–1.47)	77.1%	<0.01	11	1.32 (0.97–1.81)	72.6%	<0.01	10	0.93 (0.67–1.28)	66.9%	<0.01	10	0.63 (0.47–0.84)	59.6%	0.01
Regions	North America	13	1.47 (1.24–1.75)	26.3%	0.18	9	1.40 (1.21–1.62)	17.2%	0.29	5	1.15 (0.96–1.36)	7.2%	0.37	7	0.92 (0.81–1.05)	1.7%	0.41
	Europe	7	1.10 (0.83–1.44)	57.2%	0.03	4	0.97 (0.73–1.28)	23.9%	0.27	4	0.89(0.70–1.13)	0.0%	0.78	4	0.70 (0.55–0.89)	0.0%	0.97
	Asian and others	8	0.84 (0.56–1.27)	82.6%	<0.01	5	1.71 (0.75–3.88)	79.2%	<0.01	5	0.95 (0.40–2.23)	79.8%	<0.01	5	0.44 (0.24–0.78)	58.0%	0.05
Design	Cohort	2	1.06 (0.74–1.53)	0.0%	0.55	2	1.14 (0.88–1.48)	0.0%	0.43	2	0.95 (0.74–1.24)	0.0%	0.58	2	0.95 (0.76–1.18)	0.0%	1.00
	PBCC	18	1.23 (1.01–1.50)	71.2%	<0.01	10	1.62 (1.21–2.16)	69.8%	<0.01	7	1.26 (0.99–1.61)	34.7%	0.16	9	0.78 (0.59–1.01)	66.2%	<0.01
	HBCC	8	1.11 (0.74–1.67)	73.8%	<0.01	6	1.04 (0.82–1.31)	18.6%	0.29	5	0.69 (0.46–1.05)	58.3%	0.05	5	0.61 (0.47–0.78)	0.0%	0.50
DietaryAssessment	HHHQ	3	1.69 (1.21–2.36)	0.0%	0.46	2	1.81 (0.71–4.60)	57.8%	0.12	0				0			
	FFQ(>100 items)	12	1.10 (0.93–1.31)	39.3%	0.08	10	1.19 (1.00–1.41)	33.8%	0.14	8	1.04 (0.87–1.25)	21.0%	0.26	10	0.87 (0.77–0.98)	0.0%	0.53
	FFQ(≤ 100 items)	13	1.22 (0.88–1.70)	80.7%	<0.01	6	1.68 (0.99–2.85)	79.8%	0.00	6	1.00 (0.58–1.73)	79.8%	<0.01	6	0.53 (0.32–0.88)	77.2%	<0.01
N. (Case)	N. > 250	15	1.03 (0.85–1.26)	75.2%	<0.01	9	1.22 (0.99–1.50)	65.4%	<0.01	7	0.97 (0.73–1.29)	74.7%	<0.01	9	0.84 (0.71–1.00)	53.9%	0.03
	N. ≤ 250	13	1.53 (1.14–2.07)	53.8%	0.01	9	1.61 (1.09–2.38)	58.8%	<0.01	7	1.07 (0.70–1.63)	47.4%	0.08	7	0.58 (0.38–0.88)	48.5%	0.07
Study quality	Score≥7	17	1.03 (0.84–1.25)	71.6%	<0.01	12	1.36 (1.08–1.71)	69.2%	<0.01	10	1.06 (0.80–1.40)	70.9%	<0.01	12	0.77 (0.63–0.96)	66.6%	<0.01
	Score≤ 6	11	1.54 (1.15–1.89)	56.9%	0.01	6	1.20 (0.89–1.62)	28.8%	0.23	4	0.82 (0.61–1.10)	0.0%	0.49	4	0.70 (0.52–0.95)	0.0%	0.96

SRR, summary relative risk

CI, confidenceinterval

USA, United States of America

GCA, gastric cardia adenocarcinoma

GNCA, gastric non-cardia adenocarcinoma

NOS, Newcastle-Ottawa Scale

N., number

PBCC, Population-based case-control

HBCC, Hospital-based case-control

HHHQ, health habits and history questionnaire

FFQ, food frequency questionnaire.

We also performed subgroup analyses according to different publications years (before 2000 and after 2000) to investigate the association between total fat intake and gastric cancer risk. The results showed no significant difference between two groups (studies published before 2000: SRR = 1.33; 95% CI: 0.97–1.82;n = 12; *P*< 0.001;tau^2^ = 0.22; I^2^ = 78.3%; studies published after 2000: SRR = 1.10; 95% CI: 0.90–1.33; n = 16;*P* = 0.001; tau^2^ = 0.08; I^2^ = 60.0%). No evidence of publication bias was observed in the subgroup published after 2000, as tested using Egger’s (*P* = 0.67) and Begg’s (*P* = 1.00) tests. But obvious publication bias was observed in the subgroup published before 2000, as tested using Egger’s (P = 0.02) and Begg’s (P = 0.27) tests. The results showed that publication bias was more likely in older studies, which was in agreement with previous study [61].

**Fig 3:**
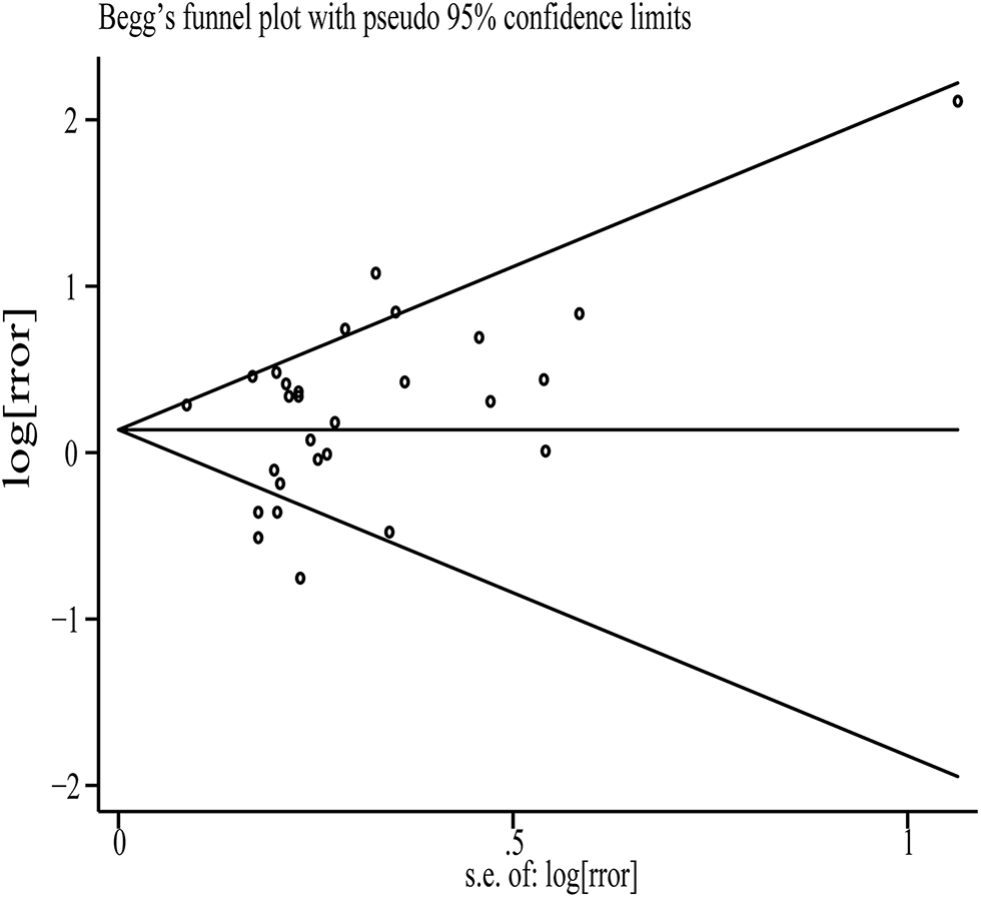
Begg’s funnel plots of studies for total fat intake and gastric cancer risk.

However, in sensitivity analysis by omitting one study each time, the SRR after exclusion one of 9 studies became statistically significant, with a range from 1.19 (95% CI: 1.002–1.41; *P*< 0.001; tau^2^ = 0.12; I^2^ = 70.5%) to 1.22 (95% CI: 1.04–1.43; *P*< 0.001; tau^2^ = 0.10; I^2^ = 64.3%). Therefore,the non-significant association between total fat intake and gastric cancer risk should be interpreted with caution. No evidence of publication bias was observed in the funnel plot, as tested using Egger’s (*P* = 0.43) and Begg’s (*P* = 0.29) tests(Fig 3).

**Fig 4:**
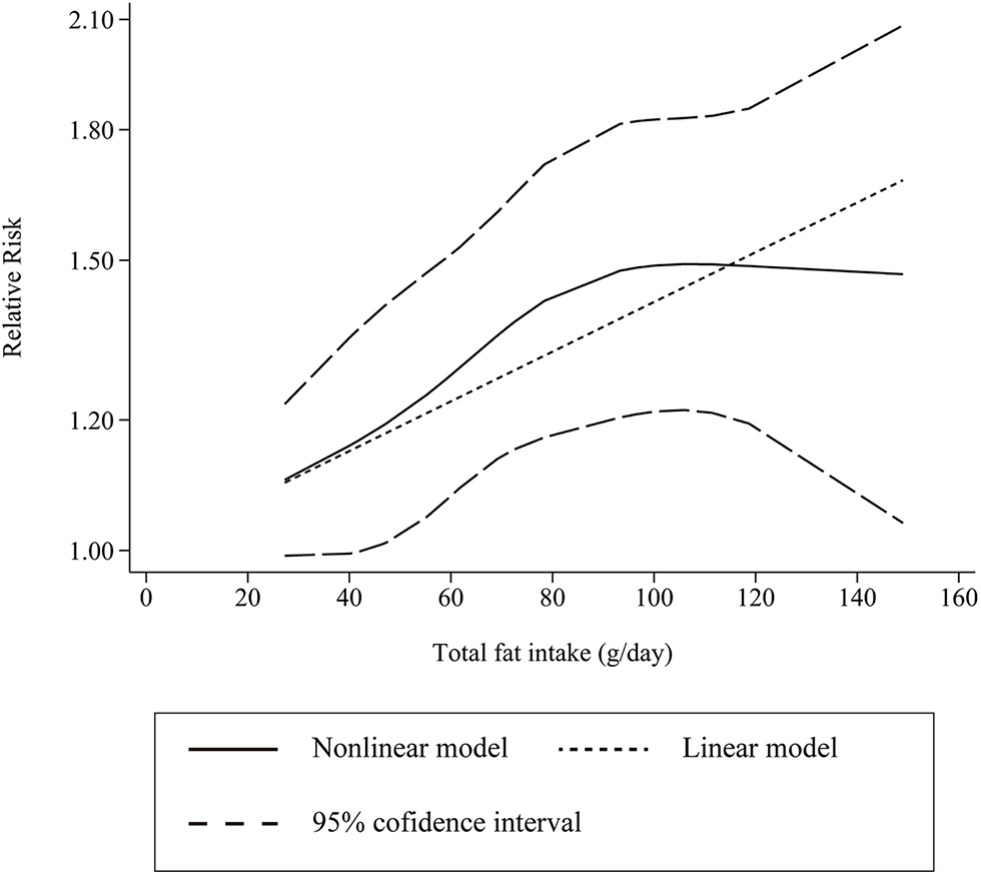
Dose-response relationship for total fat intake level and the RR of gastric cancer risk (*P*-nonlinearity = 0.50).

Unfortunately, only one cohort article (2studies) and 3case-control articles (4studies) providing the distribution of cases and controls (person-year)were eligible for the dose-response analysis. The results showed that the SRR per 20 g/d total fat increase intake was 1.08 (95%CI: 1.02–1.14), with moderate heterogeneity (*P* = 0.09; tau^2^ = 0.002;I^2^ = 46.8%, 95% CI: 0%-79%).However, the increased risk of total fat increase intake per 20 g/d was only found in 4 case-control studies(SRR = 1.12;95% CI: 1.03–1.22;*P* = 0.07; I^2^ = 57.5%)but not in 2 cohort studies (SRR = 1.03;95% CI: 0.97–1.09;*P* = 0.67; I^2^ = 0.0%).There was no evident non-linear association between total fat intake and gastric cancer risk(*P*-nonlinearity = 0.50; Fig 4).

### Saturated fat consumption

**Fig 5:**
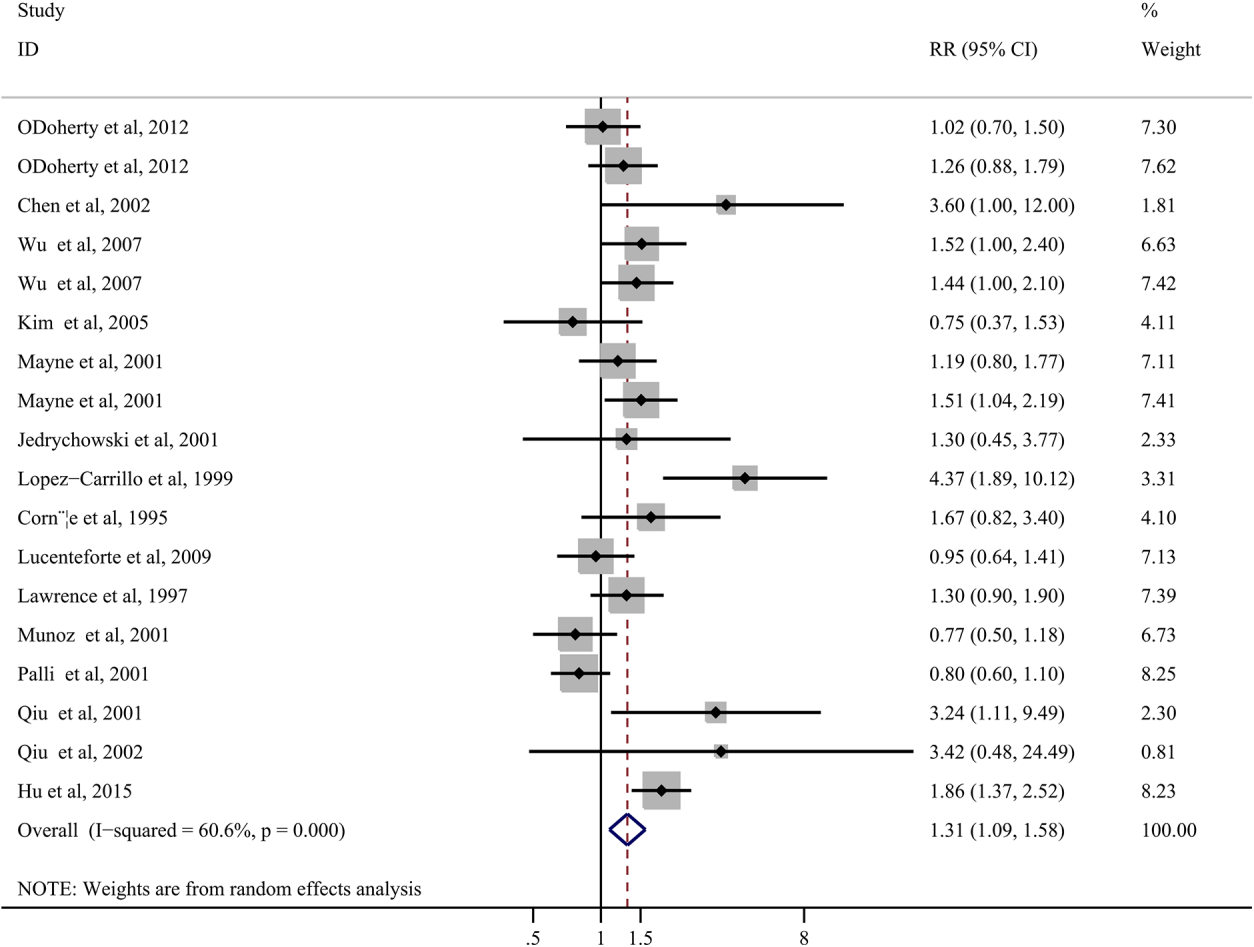
Forest plots of saturated fat intake and gastric cancer risk based on highest versus lowest analysis.

Fourteen articles with 18 studies have investigated the association between saturated fat intake and gastric cancer risk. The SRR for the highest compared with lowest analysis was1.31 (95% CI: 1.09–1.58), indicating a significant positive association(Fig 5). However, significant heterogeneity among studies was found (*P*< 0.001; tau^2^ = 0.08; I^2^ = 60.6%, 95% CI: 34%-76%).

Subgroup analyses were then applied. Significant positive association between saturated fat intake and gastric cancer risk was only found in studies of GNCA patients (SRR = 1.43; 95% CI: 1.16–1.76;n = 4; *P* = 0.44; I^2^ = 0.0%), studies located in North America (SRR = 1.40; 95% CI: 1.21–1.62; n = 9;*P* = 0.29; I^2^ = 17.2%), population-based case-control studies (SRR = 1.62; 95% CI: 1.21–2.16; n = 10;*P*< 0.001; I^2^ = 69.8%), studies with a small sample size (SRR = 1.61; 95% CI: 1.09–2.38;n = 9;*P* = 0.01; I^2^ = 58.8%), and high quality studies (SRR = 1.36; 95% CI: 1.08–1.71;n = 12;*P* = 0.00; I^2^ = 69.2%), but not in other subgroups (Table 1).

Sensitivity analysis showed that no individual study could change the summary positive association between saturated fat intake and gastric cancer risk. There was no evidence of publication bias, as tested using Egger’s(*P* = 0.15) and Begg’s (*P* = 0.34) tests. Unfortunately, only 3studies (from 2 articles) were eligible for the dose-response analysis. Thus, we did not perform dose-response analysis in this group.

### Polyunsaturated fat consumption

**Fig 6:**
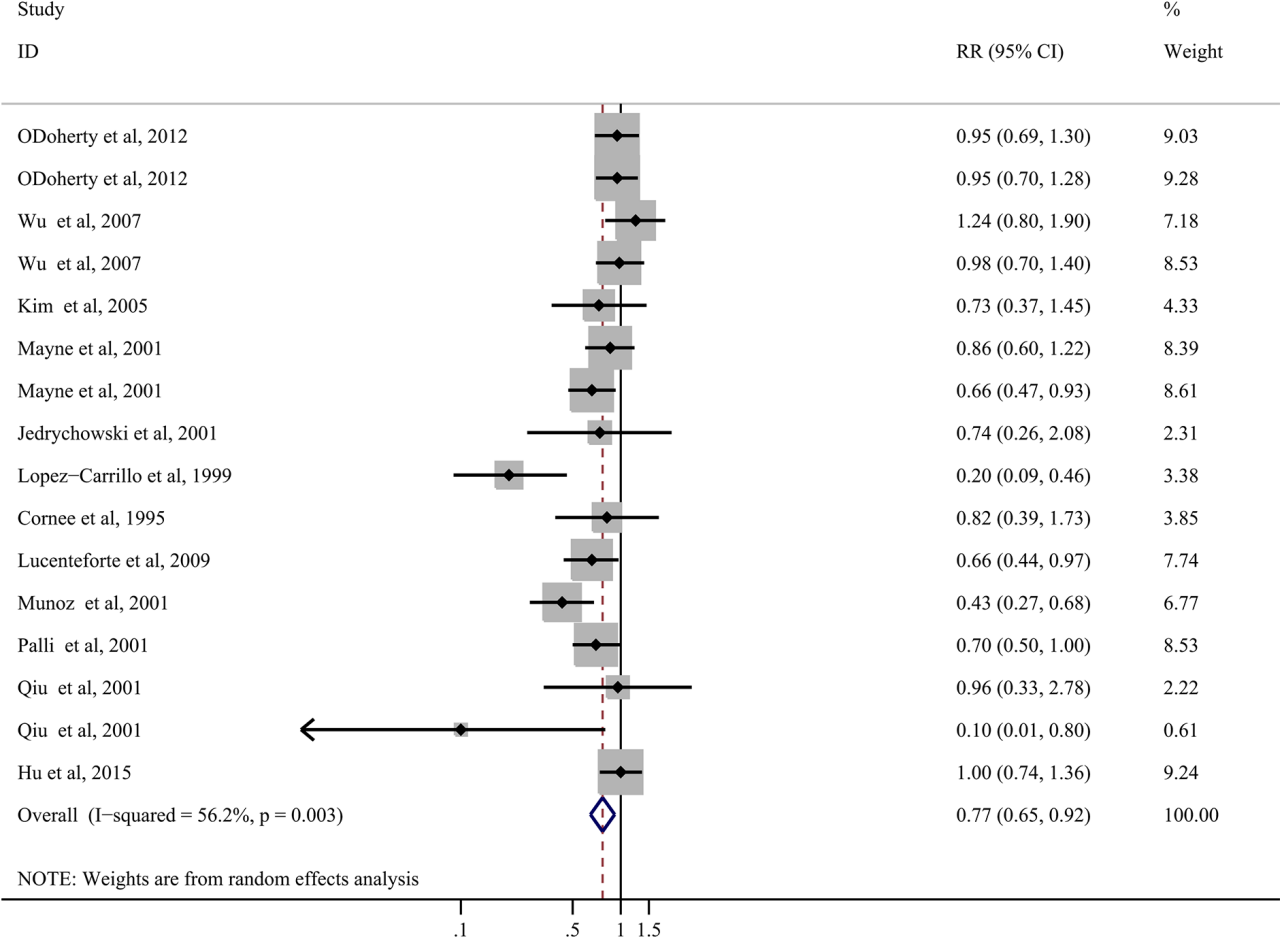
Forest plots of polyunsaturated fat intake and gastric cancer risk based on highest versus lowest analysis.

Twelve articles with 16 studies have investigated the association between polyunsaturated fat intake and gastric cancer risk. The SRR for the highest compared with lowest analysis was0.77 (95% CI: 0.65–0.92), indicating a significant inverse association. However, significant heterogeneity was detected (*P* = 0.003; tau^2^ = 0.06; I^2^ = 56.2%, 95% CI: 23%-75%; Fig 6).

In subgroup analysis, the inverse association between polyunsaturated fat intake and gastric cancer risk was no more significant when we restricted to studies of GCA patients(SRR = 0.98; 95% CI: 0.79–1.20; n = 3;*P* = 0.43; I^2^ = 0.0%) or GNCA patients only (SRR = 0.85; 95% CI: 0.67–1.09, n = 3;*P* = 0.19, I^2^ = 38.9%), studies located in North America (SRR = 0.92; 95% CI: 0.81–1.05; n = 7;*P* = 0.41, I^2^ = 1.7%), cohort studies (SRR = 0.95, 95% CI: 0.76–1.18; n = 2;*P* = 1.00, I^2^ = 0.0%), population-based case-control studies (SRR = 0.78; 95% CI: 0.59–1.01; n = 9;*P* = 0.00, I^2^ = 66.2%), and studies with a large sample size (SRR = 0.84; 95% CI: 0.71–1.00; n = 9;*P* = 0.03, I^2^ = 53.9%). In other subgroups, the summary results were consistent with the overall results of all studies (Table 1).

No individual study could change the summary results in the sensitivity analyses. No significant evidence of publication bias was detected, as tested using Egger’s(*P* = 0.11) and Begg’s (*P* = 0.06) tests. We did not perform dose-response analysis in this group due to very few eligible studies.

### Monounsaturated fat consumption

Elevenarticles with 14 studies have investigated the association between monounsaturated fat intake and gastric cancer risk. The SRR for the highest compared with lowest analysis was1.00 (95% CI: 0.79–1.25), with significant heterogeneity among studies (*P*< 0.001; tau^2^ = 0.10; I^2^ = 63.0%, 95% CI: 34%-79%).

In subgroup analyses, the results were fairly consistent with the overall summary estimates when the analyses were restricted to any subgroup(Table 1), indicating no association between monounsaturated fat intake and gastric cancer risk in any subgroup. Sensitivity analysis showed that SRR was not markedly modified by any individual study. Noevidence of publication bias was detected, as tested using Egger’s(*P* = 0.67) and Begg’s (*P* = 0.74) tests. Dose-response analysis was not performed due to too few eligible studies.

### Animal fat and vegetable fat consumption

Out of the included studies, 6studies (from 5articles)and 4studies have investigated the associations between intake of animal fat and vegetable fat and gastric cancer risk, respectively. The SRRs for the highest compared with lowest analysis were1.10 (95% CI: 0.90–1.33;*P* = 0.13;tau^2^ = 0.02; I^2^ = 42.0%, 95% CI: 0%-70%) and 0.55 (95% CI: 0.41–0.74;*P* = 0.12; tau^2^ = 0.04; I^2^ = 48.6%, 95% CI: 0%-83%), respectively. The results indicated that consumption of vegetable fat but not animal fat could reduce the risk of gastric cancer. Sensitivity analyses showed SRRs were not markedly modified by any individual study in two groups.

## Discussion

Although the result of highest versus lowest meta-analysis does not strongly support a positive association between intake of total fat and gastric cancer risk, the result of the dose-response analysis indicate that a daily increase intake of 20 g total fat is significantly associated with a8% higher risk of gastric cancer. Our highest versus lowest meta-analyses support a positive association between intake of saturated fat and gastric cancer risk,an inverse association between intake of polyunsaturated fatand vegetable fat and gastric cancer risk, and no association between intake of monounsaturated fat and animal fat and gastric cancer risk.

In subgroup analyses, we observed that high intake of total fat was positively associated with gastric cancer risk in studies conducted in North America without evident heterogeneity. The adverse effect of total fat in North America might attribute to the high fat diet habits of people living in western countries[62, 63].However, we did not observe similar effect of total fat in European countries. When we analyzed each study in European countries, 3 studies from Italy reported that the total fat tended to decrease the risk of gastric cancer (RR < 1). However, 4 studies conducted in other European countries reported that the total fat tended to increase the risk of gastric cancer (RR > 1). This indicated that the dietary structure and nutriment intake of Italian might be different from people in other western countries[64, 65]. Moreover, positive associations between consumption of total fat and gastric cancer were more common in studies with small sample size and low quality. Thus, theas sociation between total fat intake and gastric cancer risk should be interpreted with caution.

Epidemiological and experimental studies have suggested that different fatty acid play different roles in the carcinogenesis and progression of human cancers [66]. In this meta-analysis, we found different associations between specific fatty acid intake and gastric cancer risk, indicating that different fatty acid did have different effects on gastric cancer.

As for saturated fat,our results were consistent with a majority of studies which showed that high intake of saturated fat could increase the risk of malignant tumors (such as breast and colorectal cancer)[14, 67–69]. However, saturated fat was found not to be associated with some other cancers[18, 70]. In experimental studies, diets with a high content in saturated fat were demonstrated to promote the tumorigenesis[71]. Interestingly, in subgroups analysis of our meta-analysis, intake of saturated fat significantly associated with GNCA risk but not GCA risk, which was consistent with the results of total fat.The difference may partly due to the small number of studies that provide the separate data for GNCA and GCA. However, as reported before, some other risk factors for gastric cancer were not consistent across all cancer sites either[72–74]. It is now clear that two anatomic locations of stomach, cardia and non-cardia, present distinct and sometimes opposite epidemiological characteristics[75]. Therefore, future studies need to distinguish those two different anatomic locations. In addition, saturated fat was considered to be a significant risk factor for people living in North America, which was in line with the results of total fat, suggesting that saturatedfat maybe the main source of dietary total fat inNorth America[76].

The major monounsaturated fatty acid found in human diet isoleic acid. Mediterranean diet, characterized by the high consumption of olive oil, rich in oleic acid, has been traditionally linked to a protective effect on cancers [77]. In a cohort study including 485,044 subjects from 10 European countries, adherence to a relative Mediterranean diet was associated with a significant reduction ofgastric cancer risk[78]. Similar protective effect of Mediterranean diet for breast cancer and colorectal cancer was observed [79, 80]. However, the protective effect of olive oil for gastric cancer was not observed in a study focused on olive oil but not Mediterranean diet[35]. This might be interpreted by the complex composition of Mediterranean diet (such as high consumption of fruit, vegetables, fish, and seafood)[77]. In our meta-analysis, high intake of monounsaturated fat (including oleic acid) showed no significant association with gastric cancer risk, which was consistent with the results of colorectal cancer[18]. In fact, some differences exist between single chemically defined nutrients(such as oleic acid) and foodstuff (such as olive oil)[81]. Olive oil contains not only oleic acid as nutrient but also many minor compounds which were defined as “bioactivecompounds” and exerted main protective effects of cancer[82, 83]. Moreover, experimental studies have provided evidence of olive oilto influence the hormonal status, cell membranes structure and function, signal transduction pathways, gene expression and the immune system[71].

Opposite to our findings of protective effect of polyunsaturated fat on gastric cancer, polyunsaturated fat was considered to increase the risk of breast cancer and had no effect on colorectal cancer[18, 68]. It was reported that n-3 and n-6polyunsaturated fatty acids (PUFAs)may have different functions in the process of carcinogenesis. Generally, n-6 PUFAs stimulated the development of cancers whereasn-3 PUFAs may inhibit the development of cancers such as breast cancer and colorectal cancer[71]. Unfortunately, in our meta-analysis, a majority of included studies provided the overall effect of polyunsaturated fat but not n-3 PUFAs and n-6PUFAs separately. When the overall effects were combined, an inverse association was observed between polyunsaturated fat intake and gastric cancer risk. In a large systematic review of cohort studies published before 2006, which investigated the association betweenn-3 PUFAs and a variety of cancers, no consistent evidence of a reduced risk of any cancer (including gastric cancer)was observed[84]. Therefore, the author declared that dietary supplementation with n-3 PUFAswas unlikely to prevent any cancer. In the cohort study included in this meta-analysis, no association was observed between n-3 PUFAs and gastric cancer risk either. However, experimental studies have demonstrated thatn-3 PUFAs induced cell apoptosis in human colorectal cancer, breast cancer, pancreatic cancer, and prostatic cancer[85–88]. N-3 PUFAs was also demonstrated to exert an anti-cancer effect on gastric cancer by inducing apoptosis of gastric cancer cells via ADORA1[89]. In addition, n-3 PUFAs emulsion-based parenteral nutrition alleviated the inflammatory reaction and reduced the rate of inflammatory complications after gastric cancer [90].Taken together, whether n-3 PUFAs exert a protective effect on gastric cancer needs further analysis.

In this meta-analysis, we observed an inverse association between vegetable fat intake and gastric cancer risk and no association for animal fat.However, it has been shown that for small meta-analyses homogeneity is often assumed when this is not the case and existing heterogeneity is not detected [91]. In addition, the compositions of vegetable fat and animal fat are complex. Thus, the association between vegetable and animal fat intake and gastric cancer risk should be interpreted with caution.

Our meta-analysis had several strengths. As far as we known, it is the first meta-analysis focused on the association between dietary fat intake and gastric cancer risk. Our meta-analysis included a large number of studies and more than 9,000 cases, and over 500,000 non-cases in this analysis. Thus we had adequate statistical power to clarify the association between dietary fat intake and gastric cancer risk. We have also quantified the association between intake of dietary fat and gastric cancer risk by carrying out dose-response analyses and the results were generally consistent with the highest versus lowest analysis.

This meta-analysis has several limitations. First, only one cohort study was included and the rest 21 studies were case-control studies, which may introduce recall bias and selection bias, especially the hospital-based case-control studies.The associations between intake of polyunsaturated fat and saturated fat and gastric cancer risk were not statistically significant in the cohort study but are statistically significant in the case-control studies. More cohort studies are needed to confirm the association in this cohort study. Second, most of included case-control studies did not report the doses or number of cases and controls for each category. Therefore, the results of dose-response analysis were not credible because too few eligible studies were available for the dose-response meta-analysis. Future studies should report the complete results with standardized dietary fat categories and number of cases and control in order to assess the dose-response relationship. Third, a majority of studies only adjusted forage, sex, and total energy intake. Some other known risk factors for gastric cancer such as family history, BMI, and *Helicobacter pylori* infection were not adjusted in most studies. Thus, the possibility of residual confounders as potential explanation for the observed association between dietary fat intake and gastric cancer risk could not be excluded. Moreover, it could not be excluded that other nutrients or dietary components that were correlated with dietary fat might be responsible for the observed associations. Fourth, most studies used food frequency questionnaires to assess dietary fat intake. However, as reported before, measurement errors associated with the use of food frequency questionnaires may obscure associations between dietary fat intake and gastric cancer risk[92]. Fifth, highest and lowest category intake of dietary fat were differently defined in various studies. Therefore, the effect of dietary fat may be exaggerated in studies using quintile than quartile and tertile. Sixth, we assumed OR to be comparable with the RR in this study, which might introduce bias, although baseline risks are not low in our analyses [93].Finally, publication bias may be a problem in any meta-analysis. Although publication bias was not observed in this meta-analysis, some excluded studies due to the incomplete report may introduce bias. In addition, we restrict the language to English only, which may introduce language bias.

In conclusion, results from this meta-analysis indicate a possible significant positive association between total fat intake and gastric cancer risk. Consumption of saturated fat is positively associated with gastric cancer risk. Consumption of polyunsaturated fat and vegetable fat is inversely associated with gastric cancer risk. Consumption of monounsaturated fat and animal fat is not associated with gastric cancer risk. Caution is needed in interpreting these associations because the results of subgroups analyses were not always consistent. Additional well-designed cohort studies with detailed dietary assessment sand strict control of confounders are needed to confirm the associations between dietary fat intake and gastric cancer risk.

## Supporting Information

S1 Checklist PRISMA Checklist of this meta-analysis. (DOC)

S1 Table Characteristic of the studies with regard to dietary fat intake and gastric cancer risk. (DOCX)
