# Supplementary material for: Dietary Fat Intake and Risk of Gastric Cancer: A Meta-Analysis of Observational Studies
Source: PLoS One. 2015 Sep 24;10(9):e0138580. doi: 10.1371/journal.pone.0138580 (PMC4581710; doi:10.1371/journal.pone.0138580)
Supplement: S1 Table — (DOCX) [file pone.0138580.s002.docx]

**S1 Table.** Characteristic of the studies with regard to dietary fat intake and gastric cancer risk

| Author and year | Country | Controls /cohort size | Cases | Dietary Assessment-items- | NOS score | Partition | Fat subtypes | Subgroup | RR/OR (95%CI)  (highest vs. lowest) | Adjusted factors |
| --- | --- | --- | --- | --- | --- | --- | --- | --- | --- | --- |
| **Cohort study** | | | | | | | | | | |
| O’Doherty et al, 2012 | USA | 494,978 | 955 | FFQ-124 | 9 | Quintile | Total fat | GCA | 1.2 (0.7-2.05) | Age, sex, non-alcohol energy intake, BMI, education, ethnicity, alcohol, cigarette smoking, diabetes, physical activity, fruit and vegetable intake, and red meat intake. |
|  |  |  |  |  |  |  |  | GNCA | 0.96 (0.59-1.59) |  |
|  |  |  |  |  |  |  | Saturated fat | GCA | 1.02 (0.7-1.5) |  |
|  |  |  |  |  |  |  |  | GNCA | 1.26 (0.88-1.79) |  |
|  |  |  |  |  |  |  | Monounsaturated fat | GCA | 1.03 (0.71-1.51) |  |
|  |  |  |  |  |  |  |  | GNCA | 0.89 (0.62-1.28) |  |
|  |  |  |  |  |  |  | Polyunsaturated fat | GCA | 0.95 (0.69-1.3) |  |
|  |  |  |  |  |  |  |  | GNCA | 0.95 (0.7-1.28) |  |
| **Population-based case-control study** | | | | | | | | | | |
| Chen et al, 2002 | USA | 449 | 124 | HHHQ | 6 | Tertile | Total fat | GNCA | 2.3 (0.74-7.3) | Age and sex |
|  |  |  |  |  |  |  | Saturated fat | GNCA | 3.6 (1-12) |  |
| Wu et al, 2007 | USA | 1308 | 623 | FFQ-124 | 7 | Quartile | Total fat | GCA | 1.44 (0.9-2.2) | Age, sex, race, birthplace, education, smoking, BMI, reﬂux, use of vitamins and total calorie |
|  |  |  |  |  |  |  |  | GNCA | 1.51 (1-2.3) |  |
|  |  |  |  |  |  |  | Saturated fat | GCA | 1.52 (1-2.4) |  |
|  |  |  |  |  |  |  |  | GNCA | 1.44 (1-2.1) |  |
|  |  |  |  |  |  |  | Monounsaturated fat | GCA | 1.31 (0.9-2) |  |
|  |  |  |  |  |  |  |  | GNCA | 1.51 (1-2.3) |  |
|  |  |  |  |  |  |  | Polyunsaturated fat | GCA | 1.24 (0.8-1.9) |  |
|  |  |  |  |  |  |  |  | GNCA | 0.98 (0.7-1.4) |  |
| Mayne et al, 2001 | USA | 687 | 607 | FFQ-104 | 9 | Quartile | Total fat | GCA | 0.99 (0.59-1.66) | Age, sex, site, race, proxy status, income, education, usual BMI, cigarettes/day, years of consuming beer, wine, and liquor, and energy intake. |
|  |  |  |  |  |  |  |  | GNCA | 1.08 (0.67-1.74) |  |
|  |  |  |  |  |  |  | Saturated fat | GCA | 1.19 (0.8-1.77) |  |
|  |  |  |  |  |  |  |  | GNCA | 1.51 (1.04-2.19) |  |
|  |  |  |  |  |  |  | Polyunsaturated fat | GCA | 0.86 (0.6-1.22) |  |
|  |  |  |  |  |  |  |  | GNCA | 0.66 (0.47-0.93) |  |
| Terry et al, 2000 | Sweden | 815 | 258 | FFQ-63 | 7 | Quartile | Total Fat | GCA | 1.4 (0.9-2.1) | Age, sex, BMI, total energy, energy adjusted alcohol, total fruit and vegetable intake, cigarette smoking, and use of antacids. |
| López-Carrillo et al, 1999 | Mexico | 752 | 220 | FFQ-70 | 7 | Quartile | Total fat | All | 1.36 (0.54-3.43) | Age sex, total calories, chili-pepper consumption, SES, cigarette smoking, salt consumption, history of peptic ulcer, type of interview , duration of interview, place of interview |
|  |  |  |  |  |  |  | Saturated fat | All | 4.37(1.89-10.12) |  |
|  |  |  |  |  |  |  | Monounsaturated fat | All | 3.45 (1.39-8.56) |  |
|  |  |  |  |  |  |  | Polyunsaturated fat | All | 0.2 (0.09-0.46) |  |
| Ji et al, 1998 | China | 1451 | 1124 | FFQ-84 | 7 | Quartile | Total fat | Men | 0.7 (0.5-1) | Age, income, education, smoking (males only), alcohol drinking (males only) and total calories intake |
|  |  |  |  |  |  |  |  | Women | 0.6 (0.4-0.8) |  |
|  |  |  |  |  |  |  | Animal fat | Men | 1 (0.8-1.3) | Age, income, education, smoking (males only) and alcohol drinking (males only) |
|  |  |  |  |  |  |  |  | Women | 1.2 (0.9-1.7) |  |
| Hansson et al, 1994 | Sweden | 679 | 338 | FFQ-45 | 6 | Quartile | Total fat | 20 years prior to interview | 1.62 (1.09-2.39) | Age, sex and energy intake |
| Graham et al, 1990 | USA | 285 | 293 | FFQ-NA | 5 | Quartile | Total fat | Men | 2.94(1.55-5.57) | Age and SES. |
|  |  |  |  |  |  |  |  | Women | 2.33 (1.17-4.64) |  |
| Buiatti et al, 1990 | Italy | 1159 | 1016 | FFQ-146 | 7 | Quintile | Total fat | All | 0.7 (0.5-1.1) | Non-dietary variables (Age, sex, area, place of residence, migration from south, SES, familial GC history, Quetelet index) and kilocalories |
|  |  |  |  |  |  |  | Animal fat | All | 1.7 (1.2-2.5) | Non-dietary variables, kilocalories, animal fat. |
|  |  |  |  |  |  |  | Vegetable fat | All | 0.4 (0.3-0.6) | Non-dietary variables and kilocalories animal fat. |
| Risch et al, 1985 | Canada | 246 | 246 | FFQ-NA | 7 |  | Unsaturated fat | All | 2.65 (1.21-5.81) | Food consumption and ethnicity |
| Munoz et al, 2001 | Venezuela | 485 | 302 | FFQ-75 | 7 | Quartile | Total fat | All | 0.47 (0.3-0.74) | Age, sex, alcohol, tobacco, total calories and SES |
|  |  |  |  |  |  |  | Saturated fat | All | 0.77 (0.5-1.18) |  |
|  |  |  |  |  |  |  | Monounsaturated fat | All | 0.39 (0.25-0.62) |  |
|  |  |  |  |  |  |  | Polyunsaturated fat | All | 0.43 (0.27-0.68) |  |
| Palli et al, 2001 | Italy | 561 |  | FFQ-181 | 7 | Tertile | Total fat | All | 0.9 (0.6-1.3) |  |
|  |  |  |  |  |  |  | Vegetable fat | All | 0.7 (0.5-1) | Age, sex, social class, family history of gastric cancer, area of rural residence, BMI, and total energy |
|  |  |  |  |  |  |  | Animal fat | All | 1 (0.7-1.4) |  |
|  |  |  |  |  |  |  | Saturated fat | All | 0.8 (0.6-1.1) |  |
|  |  |  |  |  |  |  | Monounsaturated fat | All | 0.9 (0.6-1.2) |  |
|  |  |  |  |  |  |  | Polyunsaturated fat | All | 0.7 (0.5-1) |  |
| Qiu et al, 2001 | China | 133 | 103 | FFQ-60 | 7 | Quartile | Total fat | Men | 1.01 (0.35-2.92) | Age, present residence, education, economic status, smoking (males only), alcoholics (males only) and total calories intake |
|  |  |  |  |  |  |  |  | Women | 8.26(1.03-66.51) |  |
|  |  |  |  |  |  |  | Saturated fat | Men | 3.24 (1.11-9.49) |  |
|  |  |  |  |  |  |  |  | Women | 3.42(0.48-24.49) |  |
|  |  |  |  |  |  |  | Monounsaturated fat | Men | 1.17 (0.43-3.22) |  |
|  |  |  |  |  |  |  |  | Women | 1.32 (0.17-10.4) |  |
|  |  |  |  |  |  |  | Polyunsaturated fat | Men | 0.96 (0.33-2.78) |  |
|  |  |  |  |  |  |  |  | Women | 0.1 (0.01-0.8) |  |
| Pakseresht  et al, 2011 | Iran | 304 | 286 | FFQ-117 | 7 | NA | Total fat | All | 1.33 (1.12-1.57) | Age, sex, education, living area, smoking, gastric symptoms, income, owning refrigerator, duration of using refrigerator, seeds preparing method, frying, Helicobacter pylori infection and total energy intake |
|  |  |  |  |  |  |  |  | GCA | 1.13 (0.78-1.64) |  |
|  |  |  |  |  |  |  |  | GNCA | 1.33 (1.12-1.58) |  |
| Hu et al, 2015 | Canada | 5039 | 1181 | FFQ-69 | 8 | Quartile | Total Fat | All | 1.58 (1.13-2.2) | Age, sex, province, race/ethnicity, education, BMI, alcohol drinking, pack-year smoking, fruit and vegetables, protein, cholesterol, and energy intake |
|  |  |  |  |  |  |  | Saturated fat | All | 1.86 (1.37-2.52) |  |
|  |  |  |  |  |  |  | Monounsaturated fat | All | 1.17 (0.85-1.61) |  |
|  |  |  |  |  |  |  | Polyunsaturated fat | All | 1 (0.74-1.36) |  |
| **Hospital-based case-control study** | | | | | | | | | | |
| Kim et al, 2005 | South Korea | 136 | 136 | FFQ-109 | 5 | Tertile | Total fat | All | 0.62 (0.32-1.23) | Age, sex, SES, family history, refrigerator use , and Helicobacter pylori infection |
|  |  |  |  |  |  |  | Animal fat | All | 0.93 (0.45-1.92) |  |
|  |  |  |  |  |  |  | Vegetable fat | All | 0.49 (0.24-0.99) |  |
|  |  |  |  |  |  |  | Saturated fat | All | 0.75 (0.37-1.53) |  |
|  |  |  |  |  |  |  | Polyunsaturated fat | All | 0.73 (0.37-1.45) |  |
|  |  |  |  |  |  |  | Monounsaturated fat | All | 0.55 (0.26-1.13) |  |
| Jedrychowski et al, 2001 | Poland | 260 | 80 | FFQ-148 | 5 | Tertile | Total fat | All | 1.55 (0.54-4.47) | Education, total energy, fiber intake and occupational physical activity level. |
|  |  |  |  |  |  |  | Saturated fatty acids | All | 1.3 (0.45-3.77) |  |
|  |  |  |  |  |  |  | Monounsaturated fat | All | 1.31 (0.5-3.44) |  |
|  |  |  |  |  |  |  | Polyunsaturated fat | All | 0.74 (0.26-2.08) |  |
| Cornée et al, 1995 | France | 128 | 92 | FFQ-158 | 5 | Tertile | Total fat | All | 1.53 (0.75-3.11) | Age, sex, occupation and total energy intake |
|  |  |  |  |  |  |  | Saturated fat | All | 1.67 (0.82-3.4) |  |
|  |  |  |  |  |  |  | Monounsaturated fat | All | 1.01 (0.51-2.01) |  |
|  |  |  |  |  |  |  | Polyunsaturated fat | All | 0.82 (0.39-1.73) |  |
| Kabat et al, 1992 | USA | 4544 | 69 | FFQ-30 | 4 | Quartile | Total fat | Men  GNCA | 2 (0.8-4.8) | Age , smoking, alcohol, education, hospital, and remaining dietary factors |
| Lucenteforte et al, 2009 | Italy | 547 | 230 | FFQ-78 | 6 | Tertile | Total fat | All | 0.83 (0.56-1.25) | Year of interview, education, physical activity, BMI, tobacco smoking, family history of stomach cancer and total energy intake. |
|  |  |  |  |  |  |  | Vegetable fat | All | 0.65 (0.43-0.98) |  |
|  |  |  |  |  |  |  | Animal fat | All | 0.84 (0.57-1.24) |  |
|  |  |  |  |  |  |  | Saturated fat | All | 0.95 (0.64-1.41) |  |
|  |  |  |  |  |  |  | Monounsaturated fat | All | 0.79 (0.53-1.17) |  |
|  |  |  |  |  |  |  | Polyunsaturated fat | All | 0.66 (0.44-0.97) |  |
| Lawrence et al, 1997 | USA | 132 | 91 | HHHQ | 6 | NA | Total fat | Intestinal | 1.4 (0.9-2.2) | Age, sex, caloric intake, race, education, pack-years of smoking, alcohol drinking, and BMI. |
|  |  |  |  |  |  |  |  | Diffuse | 2.1 (1.2-3.7) |  |
|  |  |  |  |  |  |  | Saturated fat | Intestinal | 1.3 (0.9-1.9) |  |
|  |  |  |  |  |  |  |  | Diffuse | 1.8 (1.1-2.8) |  |

GCA, gastric cardia adenocarcinoma; GNCA, gastric non-cardia adenocarcinoma; NOS, Newcastle-Ottawa Scale; FFQ, food frequency questionnaire; HHHQ, health habits and history questionnaire; CI, confidence interval; RR, relative risk; OR, Odds ratio; NA: not available; BMI (kg/m^2^); SES, socio-economical status.
